# Editorial: Current trends and future management of IBD

**DOI:** 10.3389/fmed.2023.1195201

**Published:** 2023-04-13

**Authors:** María Manuela Estevinho, Triana Lobatón, Nurulamin Noor, Iago Rodríguez-Lago

**Affiliations:** ^1^Unit of Pharmacology and Therapeutics, Department of Biomedicine, Faculty of Medicine, University of Porto, Porto, Portugal; ^2^Department of Gastroenterology, Centro Hospitalar Vila Nova de Gaia/Espinho, Vila Nova de Gaia, Portugal; ^3^Department of Gastroenterology, Ghent University Hospital, Ghent, Belgium; ^4^Department of Gastroenterology, Cambridge University Hospitals NHS Foundation Trust, Cambridge, United Kingdom; ^5^Gastroenterology Department, Hospital Universitario de Galdakao, Biocruces Bizkaia Health Research Institute, Galdakao, Spain

**Keywords:** Crohn's disease, ulcerative colitis, diet, environmental factor, endoscopy, artificial intelligence

Inflammatory bowel disease (IBD) is a chronic condition affecting the gastrointestinal tract with an increasing prevalence worldwide (1). It is a complex and multifactorial disorder, involving genetic and environmental factors. Individuals with a family history of IBD are also at a higher risk (2), but genetics alone cannot explain the high incidence of IBD, indicating that environmental factors and lifestyle habits play a crucial role in the pathogenesis of the disease (3).

Diet is one of the main environmental factors that are involved in this process (4). For instance, a diet high in fat, red meat, and processed foods has been associated with an increased risk of IBD. In contrast, fruits, vegetables, and whole grains may reduce the risk of developing the disease. Several studies have also shown that consuming high levels of refined sugars and ultra-processed foods can increase the risk (5) (Vissers et al.). A deep analysis into this relationship is discussed in this Research Topic collection, where Vissers et al. discuss how these components interact with the intestinal barrier, including microbiota composition, the mucus layer and epithelium integrity, and the composition of immune cells in the lamina propria. However, while environmental and lifestyle factors are relevant in population-based studies, an ongoing area of unmet needs remains in how best to evaluate and manage those individuals at higher-risk of developing IBD and this is discussed by Spencer et al. A range of circulating and stool biomarkers have been observed in at-risk individuals who subsequently develop IBD, so additional multi-omic analysis might enable better stratification of this risk. Interestingly, diet may be regarded here as both a risk factor but also an attractive target for early intervention. Future research will help us to elucidate in whom, how, and when these strategies could be utilized to provide clinical benefit through the future prevention of disease.

Tight monitoring of disease activity, including use of several targets such as biological, endoscopic and histologic remission, have been proposed in order to potentially improve outcomes in patients with IBD (6). Moreover, endoscopy and histology have an added essential role in dysplasia surveillance in patients with ulcerative colitis or Crohn's colitis. Moreover, in recent years, artificial intelligence (AI) powered systems have further optimized accuracy in endoscopic and histologic disease assessment. Alfarone et al. summarize the most relevant technical advances in IBD endoscopy and histology. The authors describe new machine learning algorithms with white light as well as high-definition endoscopes with virtual chromoendoscopy, the latter having a key role in dysplasia surveillance, but also improving accuracy of the current endoscopic activity scores. Of interest, AI capsule endoscopy techniques have also shown promising results, improving diagnostic accuracy and shortening reviewing time. Moreover, other techniques that allow microscopic observation at the cellular or even at molecular level, such as endocytoscopy and molecular imaging respectively, are also explored in detail. Importantly, Alfarone et al. also highlight the limitations of all these new tools.

Prediction of either response or non-response to therapeutics in IBD, has for many years been an aspiration in the field of IBD. Despite much initial hope at the turn of the decade that predictive serological markers might become a part of routine clinical practice (7), the many challenges of biomarker discovery, validation and translation have been recognized in recent years (8). In this regard, Alexdottir et al. highlight promising results for a potential biomarker for monitoring early response to infliximab therapy. The authors assessed serum biomarkers of collagen degradation, collagen formation, basement membrane turnover, and T-cell activity at baseline and after 14 weeks of infliximab treatment in 63 patients with Crohn's disease. The authors clearly demonstrate that baseline levels of a serological biomarker for type IV collagen degradation were associated with response to infliximab induction therapy. Moreover, the results suggest that biomarkers of type III and VI collagen formation might be used to monitor response to infliximab, for patients who had undergone prior surgery. However, loss of response or even non-response to advanced therapies is relatively frequent, and surgery should be considered in many cases. In this Research Topic, Calvino-Suarez et al. also review the potential disease burden after colectomy in ulcerative colitis patients, with the treatment options for complications after surgery such as pouchitis. In this comprehensive review from Calvino-Suarez, important aspects regarding epidemiology, risk factors and natural history of this complication are described. In addition, challenges in diagnosis, prevention and treatment are also discussed.

As well as better prediction tools to enable more precise use of current treatments, an important further element for improved IBD care is expanding the treatment options available for patients and clinicians. In recent years, one of the most exciting changes in the paradigm of IBD care has therefore been the focus and shift toward oral, small molecule therapies, including from JAK inhibitors. Herrera-deGuise et al. provide a detailed overview of this mechanism of action, including review of pivotal clinical trial data and discuss about implications for management from potential use of JAK inhibitors in the context of both UC and Crohn's disease (CD). Importantly, the authors highlight areas of potential safety concerns, along with topics for urgent clinical research focus in the coming years.

Concerning new outcomes for IBD therapy, Ramos et al. have provided an elegant review of the definitions and scores used for clinical, biochemical, endoscopic, and histologic targets in UC, whose simultaneous attainment is classified as disease clearance. The authors highlight that the current therapeutic armamentarium allows disease clearance in up to one-third of patients, with a favorable impact on prognosis in UC if disease clearance can be achieved. This concept has gained increasing attraction and interest, particularly in clinical trials, and remains an aspirational goal for clinical practice. The authors also underline the efforts that have recently been made toward defining molecular targets for UC (Ramos et al.). Indeed, unraveling the genetic pathways that are influenced—and ultimately targeted—by therapeutics is a promising approach that may pave the way toward personalized therapy in UC. Results from trials incorporating these targets as main endpoints are awaited. Conversely, a different and less straightforward scenario is seen in CD, where due to the patchy distribution of the lesions and more limited accessibility for endoscopic assessment and sampling, histologic remission is less well-defined and not commonly used to guide clinical decision-making. Instead, the concept of clearance in CD may relate to transmural healing, which can be evaluated through cross-sectional imaging. However, an in-depth evaluation of CD histopathology and validation of histologic and imaging scores and their correlation with prognosis is still lacking.

In summary, this Research Topic collection provides a comprehensive overview on: risk factors, natural history, current medical therapies and future endoscopic techniques, that may soon be incorporated in to routine care for patients with IBD—ultimately with the aim to improve outcomes and quality of life for patients living with IBD.

## Author contributions

All authors were equally involved in drafting the manuscript and reviewing for important intellectual content. All authors contributed to the article and approved the submitted version.
